# *In silico* and *in vitro* comparative analysis of 79 *Acinetobacter baumannii* clinical isolates

**DOI:** 10.1128/spectrum.02849-24

**Published:** 2025-05-16

**Authors:** Martina Scarrone, Dann Turner, Moïra Dion, Denise Tremblay, Sylvain Moineau

**Affiliations:** 1 Département de biochimie, de microbiologie et de bio-Informatique, Faculté des sciences et de génie, Université Laval 4440, Quebec City, Quebec, Canada; 2 Institut de biologie intégrative et des systèmes (IBIS), Université Laval 4440, Quebec City, Quebec, Canada; 3 School of Applied Sciences, College of Health, Science and Society, University of the West of England 1981, , Bristol, United Kingdom; 4 Félix d’Hérelle Reference Center for Bacterial Viruses, Université Laval 4440, Quebec City, Quebec, Canada; Universidad Nacional Autonoma de Mexico-Campus Morelos, Cuernavaca, Mexico

**Keywords:** *Acinetobacter baumannii*, antimicrobial resistance (AMR), bacteriophages, phages, CRISPR-Cas, capsule loci (KL), multilocus sequence typing (MLST)

## Abstract

*Acinetobacter baumannii* is a significant nosocomial bacterial pathogen that poses a substantial infection risk due to its high resistance to antibiotics and ability to survive in hospital environments. In this study, we performed comprehensive *in silico* and *in vitro* analyses on *79 A. baumannii* clinical isolates from different geographical locations to uncover their genomic and epidemiological characteristics as well as their antibiotic and phage susceptibilities. Our findings revealed considerable genomic diversity among the isolates, as shown by average nucleotide identity (ANI) heat maps, multilocus sequence typing (MLST), and core genome MLST (cgMLST). We identified several international clones known for their high antibiotic resistance and global prevalence. Surprisingly, we also observed that the number of antimicrobial resistance genes (ARGs) was higher in isolates containing CRISPR-Cas systems. Plaque assays with 13 phages indicated that *Acinetobacter* phages have a narrow host range, with capsule loci (KL) serving as a good indicator of phage-bacteria interactions. The presence of CRISPR-Cas systems and other antiviral defense mechanisms in *A. baumannii* genomes also appears to play a key role in providing phage resistance, regardless of the phage receptors. We also found that spacers associated with subtypes I-F1 and I-F2 CRISPR-Cas systems predominantly target prophages, suggesting a role in maintaining genomic stability and contributing to phage-bacteria co-evolution. Overall, this study provides a set of highly characterized *A. baumannii* clinical isolates for future studies on antibiotic-phage-bacteria interactions.

**IMPORTANCE:**

*Acinetobacter baumannii* poses a significant challenge to the healthcare system due to its antibiotic resistance and strong survival mechanisms. This study examines a diverse collection of 79 clinical isolates to deepen our understanding of *A. baumannii*’s genetic characteristics and its defense mechanisms against both antibiotics and phages. Genomic analysis revealed globally prevalent, highly resistant clones and uncovered a complex role for CRISPR-Cas systems. Although CRISPR-Cas systems were not widespread among these isolates, they primarily targeted prophages. Additionally, the study emphasizes the importance of capsule types as indicators of phage susceptibility. Together, these findings provide insights into the pathogen’s resilience and evolutionary adaptations, potentially guiding future research on infection control strategies and new therapeutic approaches to combat *A. baumannii* infections.

## INTRODUCTION

*Acinetobacter baumannii* is an opportunistic bacterial pathogen known to cause fatal nosocomial infections worldwide (1). It has been ranked as a critical priority for the development of therapeutics by the World Health Organization due to its intrinsic high resistance to several antibiotics and its ability to rapidly acquire resistance to those in clinical use (2–4). *A. baumannii* is commonly detected and isolated from hospital environments and intensive care units, causing infections such as bacteremia, ventilator-associated pneumonia, endocarditis, meningitis, urinary tract infections, and wound infections (5), though environmental and animal reservoirs have also been identified (6, 7).

A wide range of virulence factors contribute to the pathogenesis and elevated mortality associated with *A. baumannii* infections, including outer membrane proteins, lipopolysaccharides, capsular polysaccharides (CPS), iron-acquisition systems, efflux pumps, and biofilm production (8, 9). These virulence factors allow the evasion of the immune system and play a role in the survival of the pathogen and its antimicrobial resistance (AMR) (8, 10). The cell-surface CPS is one of the key determinants in *A. baumannii* as it plays a central role in its ability to cause disease, evade host defenses, and persist in hospital environments. Highly diverse CPS structures have been observed due to the polymorphism of the capsule loci (KL), accounting for at least 237 Kl identified so far in *A. baumannii* (11–13). CPS typing is also of interest because it represents a target for therapies, including antibody-based approaches or bacteriophages (phage therapy) (13, 14).

Multilocus sequence typing (MLST) is another powerful strain characterization tool that has allowed the population and epidemiology studies of a variety of pathogens, including *A. baumannii* (15). However, this approach has limitations as a phylogeny inferred from MLST fails to represent the true genomic relationships within the same bacterial species (16, 17). Core genome MLST (cgMLST) is a more extensive tool that encompasses a larger set of gene loci (hundreds to thousands instead of six to seven for MLST), enabling high-resolution bacterial genotyping (18).

A few studies have started to address the associations between the various capsule and sequence types along with the presence of carbapenem resistance genes (19–22). This information could be key for tracking the spread of this pathogen. Yet, little is known about the relationship between these features and whether there are additional underlying relationships with AMR. For example, the presence of defense systems, such as CRISPR-Cas systems, which target bacteriophages and plasmids, could restrict AMR acquisition through horizontal gene transfer (23). So far, two CRISPR-Cas systems (subtypes I-F1 and I-F2) have been predominantly identified in *A. baumannii*, though their prevalence was estimated at only 14% among nearly 5,000 isolates (24). This underscores the need to explore other defense systems as well.

Here, we performed comprehensive *in silico* and *in vitro* analyses of 79 clinical isolates of *A. baumannii*. We examined genomic diversity, KL typing, sequence types (STs), antibiotic resistance profiles, phage resistance, and antiviral defense systems. Our findings revealed significant genomic variation and key international clones linked to high antibiotic resistance. We explored the complexity of phage susceptibility, where KL typing serves as a useful predictor of phage infection, albeit it is likely influenced by various antiviral defense systems. Additionally, we found that CRISPR spacers in our set of *A. baumannii* isolates predominantly target prophages, suggesting a role in genomic stability and evolution. These insights underscore the potential for targeted therapeutic strategies, including phage therapy and novel approaches to disrupt CPS structures.

## MATERIALS AND METHODS

### Bacterial isolates, phages, and media

A total of 79 *A. baumannii* clinical isolates were obtained from various sources worldwide, and they are listed in Table S1. They were cultured with agitation at 37°C in Luria-Bertani broth (LB) (Fisher, Tryptone 10 g/L, yeast extract 5 g/L, sodium chloride 10 g/L). Thirteen *A. baumannii* phages were also used in this study, and their conditions of propagation are described in Table S2. Biological materials were stored at −80°C with 15% (vol/vol) glycerol.

### Bacterial genomic DNA extraction, sequencing, and assembly

Bacterial genomic DNA was extracted from the 79 *A*. *baumannii* isolates following a phenol-chloroform protocol (25). Illumina DNA Prep kit and Integrated DNA Technologies 10 bp unique dual indexes were used for library preparation prior to genome sequencing. Paired-end reads (2 × 151 bp) were generated with an Illumina NovaSeq 6000 sequencer (SeqCenter, Pittsburgh, PA, USA). Demultiplexing, quality control, and adapter trimming were performed with bcl-convert1 (v4.1.5). Assembly was performed with both Spades v3.13.0 (26) and Ray v3.0.1-rc (27), and the assemblies with the best coverage were kept. Genome completeness and contamination levels were calculated using CheckM2 (28).

### Genome similarity analyses

Average nucleotide identity (ANI) was calculated using FastANI with default settings (K-mer size = 16, fragment length = 3,000). The ANI results were visualized as a hierarchically clustered heatmap using the scipy and seaborn libraries in Python. For the cgMLST analysis, the *A. baumannii* schema that uses 2,390 loci (version 30/05/2021 [29]) was downloaded from www.cgMLST.org and adapted for use with chewBBACA (30), using the PrepExternalSchema module. The cgMLST clustering analysis was performed using ReporTree v2.5.4, implementing the MSTreeV2 method from GrapeTree v1.5.0, with clusters of closely related isolates being determined at ≤9 allelic differences (31). The minimum spanning tree was constructed and visualized using GrapeTree v1.5.0 (32). A maximum likelihood phylogenetic tree was inferred using IQTree2 version 2.3.4 (33) with the best fit model predicted by ModelFinder (34) using the concatenated MAFFT alignment of the set of 577 core loci from the chewBBACA analysis. Branch supports were obtained using 1000 Ultra-fast bootstrap (35) and SH-Alrt test replicates. The tree was visualized and annotated with metadata using iTOL (36).

### Multilocus sequence typing and capsular polysaccharide typing

The public database for molecular typing (PubMLST) web software (37) was used to determine the MLST profile and ST of the 79 isolates using the whole genome sequencing(WGS) assemblies and according to the Pasteur scheme (38). Whenever the results did not show a 100% identity for each allele to determine the ST accurately, a PCR was performed (38), followed by Sanger sequencing to confirm the allele. CPS typing was performed with Kaptive v. 2.0.0 using the updated *A. baumannii* KL reference sequence database (13), which was downloaded from https://github.com/katholt/Kaptive (accessed in December 2023). The KL was assigned to an isolate if the match confidence was predicted as good or higher.

### Antimicrobial susceptibility *in vitro* and antibiotic-resistance genes *in silico*

Susceptibility of the 79 isolates to 17 clinically approved antibiotics was tested *in vitro* by disk diffusion or minimal inhibitory concentration (MIC) test following the guidelines recommended by the Clinical and Laboratory Standards Institute (CLSI) (39) with 16 commercial antimicrobial disks of ceftazidime, imipenem, doripenem, tetracycline, ciprofloxacin, sulfamethoxazole/trimethoprim, piperacillin (Hardy Diagnostics, Santa Maria, CA, USA), ampicillin-sulbactam, cefepime, cefotaxime, meropenem, tobramycin, gentamicin, amikacin, minocycline, and levofloxacin (BD, Quebec City, QC, Canada). The 17th antibiotic, colistin sulfate (Sigma-Aldrich, St. Louis, MO, USA), was tested with the MIC test. Quality controls were performed for every antibiotic with *Escherichia coli* ATCC 25922. Genes mediating antimicrobial resistance from *A. baumannii* draft genomes were identified with the web server ResFinder v 4.5.0 using default thresholds of ≥80% coverage and ≥60% identity (40).

### Transmission electron microscopy

Phage morphology was confirmed or identified by transmission electron microscopy (TEM) as previously described (41) and using phosphotungstic acid-stained phage preparations.

### Phage host range determination

For the *in vitro* phage sensitivity assay, the 79 bacterial isolates were challenged with the lysates (>10^6^ PFU/mL) of 13 phages using standard spot test assays (*n* = 2) (42). Briefly, a top layer of LB with 0.75% (wt/vol) agar (supplemented with 10 mM CaCl_2_ when needed) inoculated with 100 µL of bacterial overnight culture was added to plates containing a bottom layer of LB with 1.5% (wt/vol) agar. A 5 µL volume of serial 10-fold phage dilutions (50 mM Tris-HCl, pH 7.5, 100 mM NaCl, and 8 mM MgSO_4_) was spotted onto the bacterial overlay, and the plates were incubated at 37°C overnight. A bacterial isolate was considered susceptible to a phage when the lysis zone was observed at least at dilution 10^−2^. As controls, phages were spotted onto their propagating hosts.

### Phage defense systems

Analysis on the antiviral defense systems of the 79 bacterial isolates was performed on their genome sequences using the software PADLOC v2.0.0 with models v2.0.0 (43). Candidate defense systems that have not been validated experimentally in other bacterial species were excluded from the results.

### CRISPR-Cas systems predictions and CRISPR array analysis

Identification of CRISPR-Cas systems and their arrays was performed with the web versions of both CRISPRCasFinder (44, 45) and CRISPRDetect (46) (accessed in February 2024) with default parameters. Both outputs were manually compared and curated to meet the cut-off criteria for a predicted valid array: 28 bp long direct repeats (DR) with at least 85% identity within the same array, and evidence level 4 or higher in CRISPRCasFinder. A colony PCR was also performed to confirm the presence of separate CRISPR arrays using the following specific primers: 5′-GGATTTCCTGCAATGACGGC-3′ (forward) and 5′-TCTAAAGCATCACGCCCCTG-3′ (reverse) for I-F1 and 5′-CCTGATCTTGTGCTTGGCGA-3′(forward) and 5′-GTGGTGCCGTGTTTATTGGT-3′ (reverse) for I-F2. CRISPRStudio (47) was used to produce a color-coded figure of the CRISPR arrays with default parameters, using as inputs the curated CRISPRDetect GFF format outputs. Spacer analyses were conducted in a Jupyter notebook using the Python 3.10.6 packages pandas and matplotlib.pyplot. Three databases were queried for the presence of spacer targets (protospacers) using blastn v 2.12.0 (48): the NCBI Virus database (filtered for Virus; bacteriophage, downloaded on 15 April 2024), the plasmid database PLSDB v 2023_11_03_v2 (49, 50) (downloaded on 15 April 2024), and a comprehensive database of 2,055 prophage sequences from 450 *A*. *baumannii* complete genomes (D. Turner, unpublished data). A protospacer was considered a target when the alignment showed at most four mismatches on the full length of the spacer and zero gaps. For correlation analyses, violin plots were generated using the aforementioned Python 3.10.6 packages, and Mann-Whitney U from scipy.stats and seaborn.

## RESULTS AND DISCUSSION

### Genomic data, phylogeny, MLST, and cgMLST of 79 *A. baumannii* isolates

**Fig 1:**
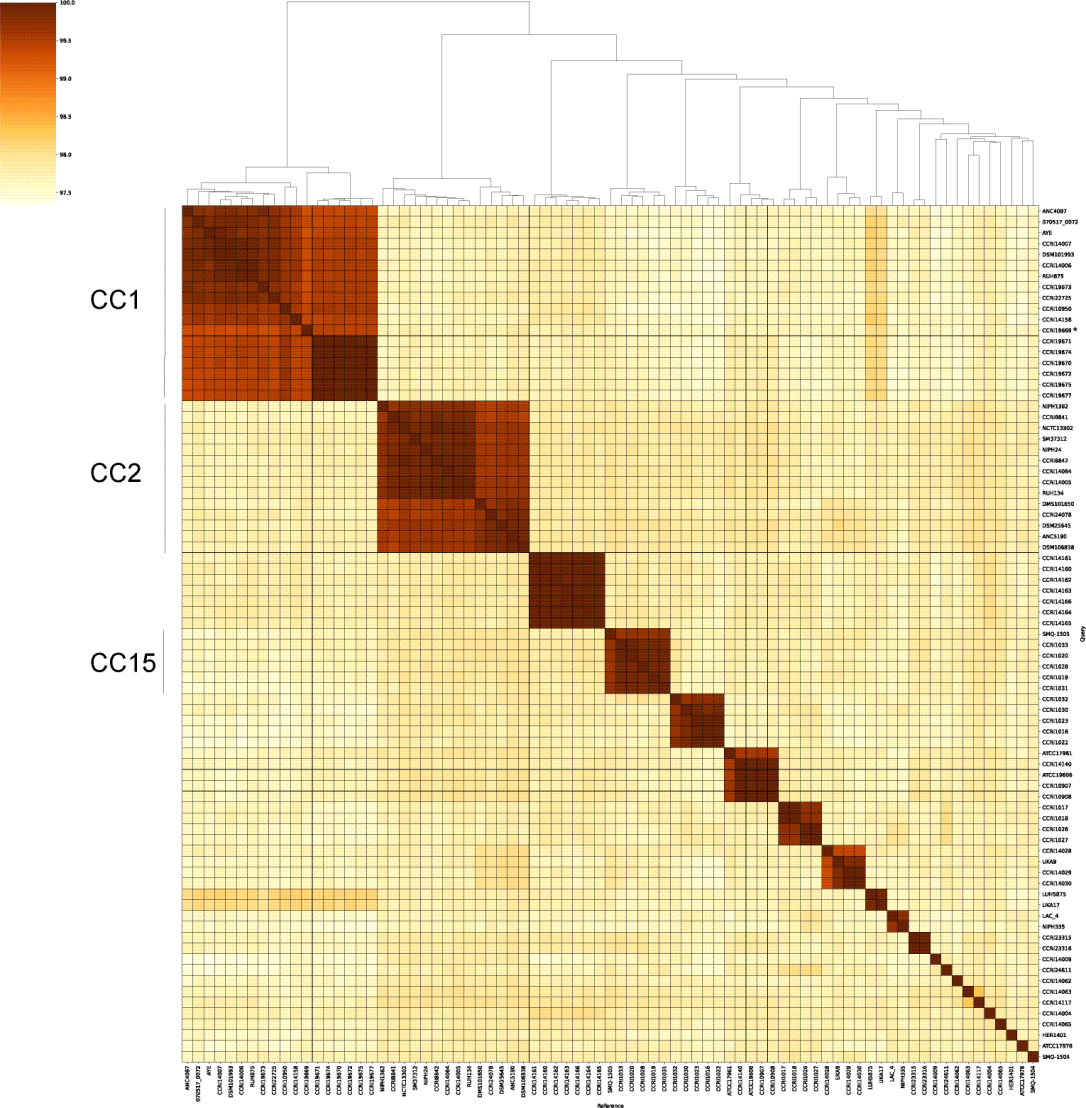
Genomic comparison of 79 *A*. *baumannii* isolates. Heat map of the average nucleotide identity (ANI). ANI values were determined using FastANI, and the heatmap was hierarchically clustered using the complete linkage method. Clonal complexes CC1, CC2, and CC15 are indicated on the left. *, isolate that does not belong to CC1.

A total of 79 *A*. *baumannii* isolates were obtained from institutes around the world, with the aim of generating a diverse collection. Genomic DNA extraction, sequencing, and assembly were performed for all isolates. Confirmation of the bacterial species was done by identification of the *bla-OXA-51-like* gene, which is intrinsic to *A. baumannii* (51). The average coverage for the draft genomes was 43.7X ± 12.4, and the average GC content was 39.0% ± 0.1 %. An ANI heat map was created to facilitate the genomic comparison of the bacterial isolates and to identify relationships (Fig. 1). The heatmap revealed significant clustering among the isolates, indicating a high degree of genomic similarity between certain groups. As an example, the upper left cluster shows six isolates (CCRI 19670, CCRI 19671, CCRI 19672, CCRI 19674, CCRI 19675, and CCRI 19677) with ANI values ≥99.9% (dark red in Fig. 1), which suggests that they are highly related. A similar clustering was observed with other isolates as well (e.g., CCRI 14160, CCRI 14161, CCRI 14162, CCRI 14163, CCRI 14164, CCRI 14165, and CCRI 14166). Conversely, the heatmap also highlighted genomic diversity among other isolates, as evidenced by overall ANI values ranging between 97.3% and 99.9 %. These more distant evolutionary relationships among these bacterial isolates (52) are likely explained by the different sources and geographical locations they were isolated (Table S1).

**Fig 2:**
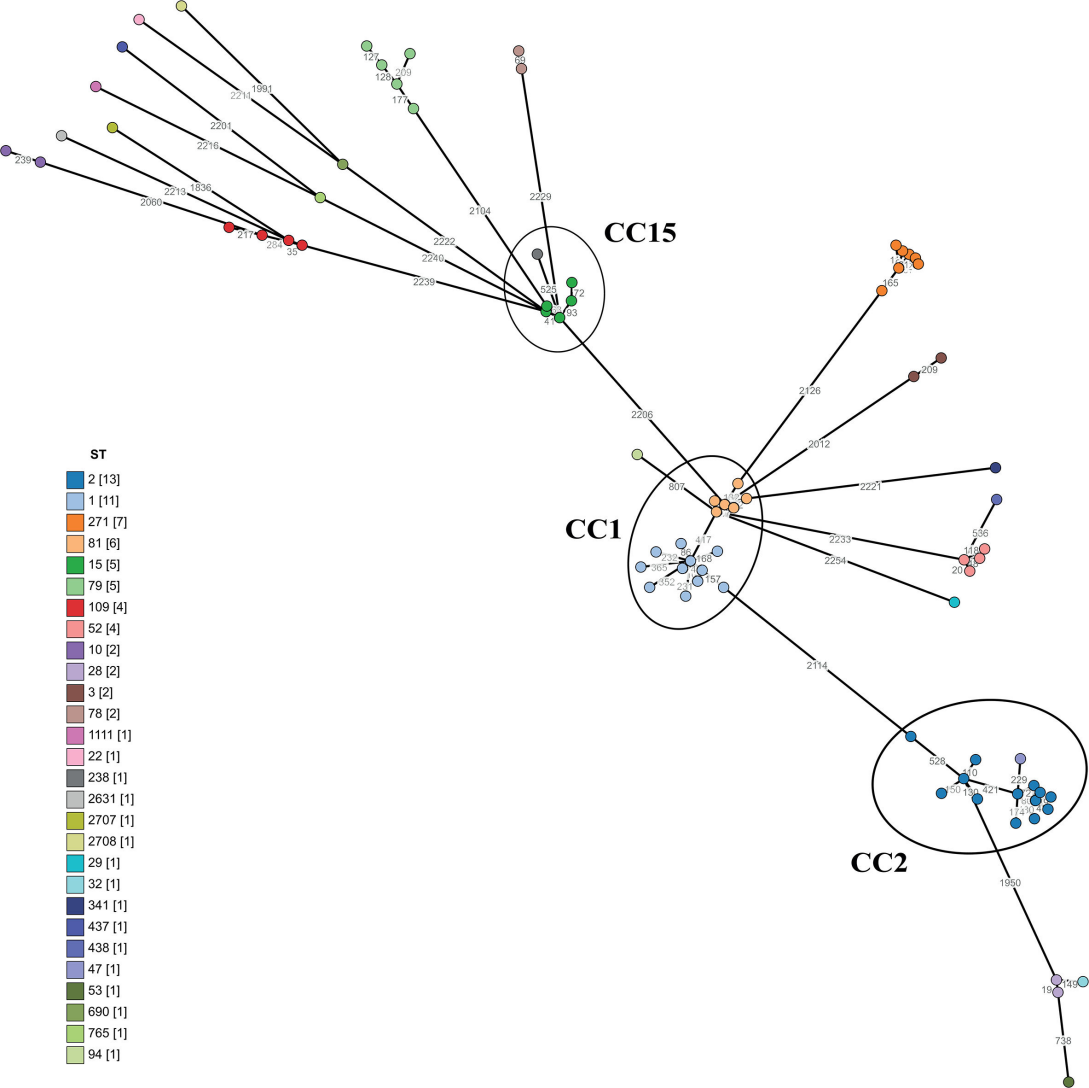
Minimum spanning tree analysis of 79 *A*. *baumannii* isolates. Representation of the genetic relationships among the cgMLST profiles of the 79 *A*. *baumannii* isolates. Each circle (node) in the tree corresponds to an individual cgMLST profile, colored according to the sequence type (ST, Pasteur scheme) as indicated in the legend, with the number of isolates shown in brackets. The edges connecting the nodes indicate genetic distances between profiles, with shorter edges representing smaller genetic differences. The numbers on the edges denote the number of allelic differences out of 2,390 alleles. CC1, CC2, and CC15 are circled, each comprising STs that share a single allelic mismatch according to the MLST Pasteur scheme (seven alleles).

To further study the genetic relationships among the 79 bacterial isolates, a minimum spanning tree (MST) was created for the cgMLST (Fig. 2), which allows for high-resolution genotyping. An ST was assigned to each isolate according to their MLST profile (Pasteur scheme), and they were subsequently grouped into a given clonal complex (CC), both informative of the population structure, distribution, and epidemiology (53). We observed a great diversity in terms of the phylogenetic lineages. Twenty-eight STs were identified, with ST1 (11/79) and ST2 (13/79) being the two most predominant. ST1 and ST2 are known to belong to the CC1 and CC2, respectively, two globally distributed lineages comprising carbapenem-resistant strains of high risk (38, 54, 55). The CC1 included ST1 and ST81 (also represented in the first upper left cluster square in Fig. 1). When we explored the geographical origin of the isolates (Table S1), we observed that all ST81 isolates, originating from Canada, were highly related (Fig. 1). For ST1, most isolates came from Europe, including Belgium (1), the Czech Republic (1), France (2), and the Netherlands (4), while others were isolated in North America (Canada, 1) and South America (Argentina, 1), with one isolate of unknown origin. A similar pattern was observed for CC2 (the second upper left cluster square in Fig. 1), composed of ST2 and ST47 isolates. Most ST2 isolates originated from Europe, including the Czech Republic (2), Georgia (1), Germany (2), Italy (1), the Netherlands (2), Spain (1), and the United Kingdom (1). One isolate was from North America (Canada), and two had no geographical information. The single ST47 isolate originated from the Czech Republic. Lastly, CC15, which includes isolates from ST15 and ST238, comprised five ST15 isolates from Argentina and one ST238 isolate from Canada. CC15 has previously been reported as a successful clonal complex in South America (56, 57).

**Fig 3:**
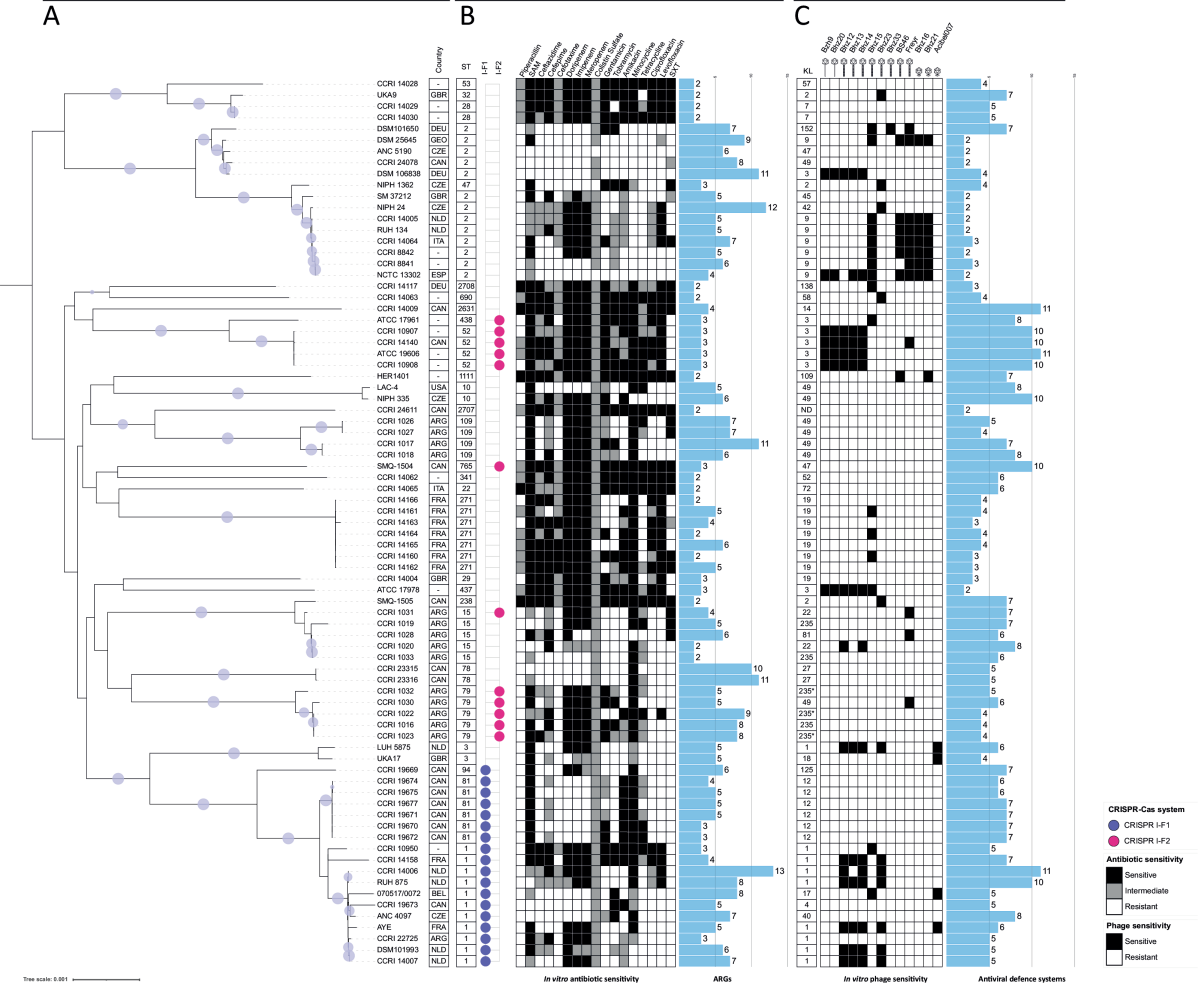
Comparative analysis of 79 *A*. *baumannii* isolates. (**A**) The maximum likelihood phylogenetic tree was inferred from the concatenated alignment of a set of 577 core loci. The tree is rooted at the midpoint, and ultra-fast bootstrap support ≥95% is shown as filled circles. The country of origin of each *A. baumannii* isolate, when available, is shown adjacent to the leaf labels as a three-letter country code: ARG (Argentina), BEL (Belgium), CAN (Canada), CZE (Czech Republic), DEU (Germany), ESP (Spain), FRA (France), GBR (United Kingdom), GEO (Georgia), ITA (Italy), NLD (Netherlands), and USA (United States). Additionally, the designated ST and the presence of subtype I-F1 (purple dot) or subtype I-F2 (fuchsia dot) CRISPR-Cas systems are indicated. (**B**) Susceptibility of the *A. baumannii* isolates to 17 antibiotics is shown as a heat map, colored according to the key on the right side of the figure. The bar chart denotes the predicted number of antimicrobial resistance genes (ARGs) from the *in silico* analysis. (**C**) The designated KL for each *A. baumannii* isolate is shown, as well as the sensitivity to 13 phages as a heat map, colored according to the key also on the right. The bar charts denote the predicted number of phage defense systems from the *in silico* analysis. *, low confidence of prediction; ND, not determined.

A maximum likelihood phylogenetic tree was also created to study the evolutionary relationships and ancestral lineages among the isolates and to compare it to their STs (Fig. 3A). Expectedly, bacterial isolates clustered in a similar pattern as shown in the ANI heat map and the MST tree for cgMLST (Fig. 1 and 2). However, we could observe the differences in evolutionary history among isolates belonging to the same ST. This was particularly evident for ST2, where the main ancestral branch encompassing all ST2 isolates splits into two distinct groups, highlighting differences in their evolutionary trajectories.

### *In silico* and *in vitro* relationship between ST and AMR

The association between the ST and AMR profiles of the isolates can provide a better understanding of the population structure and epidemiology of this pathogen (58). We assessed the antimicrobial resistance *in vitro* against 17 clinically approved antibiotics for human use (Fig. 3B; Table S1). The antibiotics that proved to be most effective were ampicillin-sulbactam and minocycline, successful against 70/79 and 62/79 isolates, respectively. On the other hand, isolates were more resistant to piperacillin and sulfamethoxazole/trimethoprim, as 21/79 and 25/79 were sensitive to these antibiotics. These results agree with some of the previous reports on *A. baumannii* clinical isolates (29, 59, 60).

We observed widely diverse resistance profiles (average of 7.0 ± 5.0 resistances per isolate). Eleven isolates were sensitive to all the antibiotics, while others showed resistance to all antibiotics of a specific class, with one isolate being resistant to all 17 antibiotics under our laboratory conditions (ANC 5190, ST2). Specific STs were resistant to more antibiotics, notably ST1, ST2, ST15, ST78, ST84, and ST91. This is in line with previous studies, where CC1 (which comprises ST1, ST84, and ST91 in this study), CC2 (ST2), and ST15 also showed multidrug resistance (29). ST2 is well recognized as a major cause of outbreaks and is associated with higher disease severity and mortality (61).

We also predicted the number of AMR genes (ARGs) in each assembled genome (Fig. 3B; Table S3). The average number of ARGs per isolate was 5.0 ± 2.7, with the cephalosporinase-encoding gene *blaADC-25* being present in 78/79 isolates. Only the isolate NCTC 13302 did not carry that gene. This analysis only comprised the acquired ARGs and does not consider possible mutations that may have a direct or indirect impact on AMR. In some cases, the number of AMR *in silico* and *in vitro* seemed to correlate well (the more AMR *in vitro*, the more ARGs), but no significant statistical correlation was observed when all isolates were compared (Pearson coefficient: −0.16). Of note, the *in silico* analysis also included genes conferring resistance to antibiotics that are not clinically approved for human use (as per the CLSI guidelines) and were therefore not tested *in vitro*.

### Capsule loci, phage host range, and antiviral defense systems

The CPS is one of the main virulence factors in *A. baumannii* and frequently serves as a receptor to initiate phage infection (62–64). The KL, which contains the genomic information to produce the CPS, was predicted with good to perfect confidence for most of the isolates (75/79, Fig. 3C). Three isolates (CCRI 1022, CCRI 1023, and CCRI 1032) had a low confidence prediction, which is likely attributed to draft genome assemblies. One isolate (CCRI 24611) showed no confidence of prediction, which may indicate the presence of a new KL not reported in the database. In total, there were 28 different KL (and one not determined), with KL1 being the most predominant (nine isolates), followed by KL49 (8), KL19 (8), KL3 (7), and KL9 (7). The different KLs did not cluster according to the phylogeny as did the STs (Fig. 3A). Thus, isolates belonging to the same KL can belong to different lineages.

The 79 *A*. *baumannii* isolates were challenged with 13 phages representing three morphotypes (Fig. 3C): two siphoviruses (long non-contractile tails), eight myoviruses (long contractile tails), and three podoviruses (short non-contractile tails). These phages, along with their bacterial hosts, were sourced from various laboratories across the globe to ensure the inclusion of the three most common *Caudoviricetes* morphotypes in our set of phages. Virion morphologies were confirmed by TEM (Fig. S1), and further information on these phages, their original hosts, and propagation conditions can be found in Table S2. Most *A. baumannii* isolates were resistant to all or most phages, and thus, these *A. baumannii* phages have a narrow host range. Each phage infected between 1 and 16 isolates, with phage Bhz33 having the smallest host range (1) and Bhz15 the broadest (16). In total, the set of phages infected 39 *A*. *baumannii* isolates. Generally, the phage host range correlated well with the KL typing, regardless of phage morphology. Several phages were capable of infecting more than one KL, usually ranging from 1 to 4 KL. Interestingly, two myophages, Freyr and Bhz15, were able to infect six and seven KL types, respectively, although this infection capability did not extend to all isolates within each KL type.

Since the CPS can be required to initiate a successful phage infection, KL may represent a key predictor of host recognition. However, the presence of intracellular defense systems could still block the phage infection process and affect the host range, even when there is phage adsorption (65). Using *in silico* analyses, the average number of defense systems per isolate was estimated at 5.5 ± 2.5 (Fig. 3C). Fifty-one systems were identified across the isolates, most of which were found in only one or a few (Table S4). The DMS_other and Mokosh type II antiviral defense systems appeared in 38 and 37 isolates, respectively. However, the PADLOC category DMS_other includes antiphage systems that may be incomplete and may not represent a distinct system. Of note, we found no statistical significance between the *in vitro* phage resistance and the *in silico* number of defense systems (Pearson coefficient: 0.059).

When comparing antiviral defense systems among the isolates within the same KL group, there was a difference in the number of systems per isolate. Taking the seven isolates of the KL3 group as an example, the antiviral defense systems ranged from 2 to 11 per isolate. Interestingly, we observed that one KL3 isolate (ATCC 17961) was sensitive to only one phage instead of five phages for the others. When we compared the type of antiviral defense systems (Table S4), ATCC 17961 was predicted to have six antiviral defense systems in common with other isolates of the same KL, as well as two unique ones: PD-Lambda-2 and cbass_type_II (66, 67). The presence of these additional systems may explain the difference observed in phage susceptibility.

Taken altogether, these findings suggest that predicting the success of the phage-bacteria interaction in *A. baumannii* will require a more comprehensive analysis. However, the association between KL type and phage specificity represents a good start.

### Presence of CRISPR-Cas systems in *A. baumannii*

A total of 29 of the 79 *A*. *baumannii* isolates (36.7%, Fig. 3A; Table S5) were found to possess a CRISPR-Cas system based on CRISPRCasFinder and CRISPRDetect tools. Two subtypes were represented (68): I-F1 (in 18 isolates) and I-F2 (in 11 isolates), which correlates with previous findings*,* in which the prevalence of the I-F1 subtype was higher in *A. baumannii* (24, 69). Their presence and subtyping were also confirmed by detecting the genes *csy2* (I-F1) and *cas7f2* (I-F2) by PCR. Notably, two separate CRISPR arrays were detected in five isolates that contained the I-F2 subtype.

We identified an association between certain STs and the presence of CRISPR-Cas systems I-F1 and I-F2 (Fig. 3A), as previously reported (24). The subtype I-F1 was observed in all ST1 and ST81 isolates (CC1) and in the only ST94 isolate, which is closely related to ST1 and ST81 in terms of phylogeny and genomic similarity (Fig. 1 and 2). As for the subtype I-F2, it was observed in all isolates assigned to ST79 and ST52. Subtype I-F2 was also seen in the only ST438 (belonging to CC52 along with the ST52) and ST765 isolates.

### CRISPR spacers predominantly target *A. baumannii* prophages

**Fig 4:**
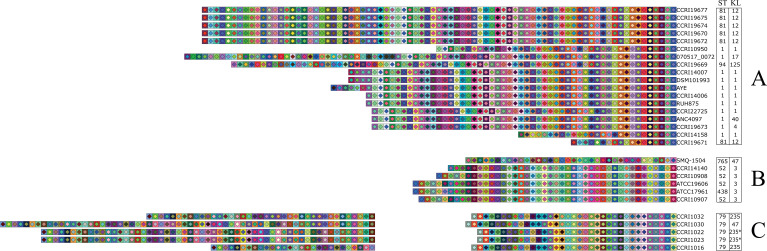
Schematic representation of spacers from CRISPR arrays in *A. baumannii* isolates. Each colored square corresponds to a spacer, ordered from 5′ to 3′ end. Two spacers with the same diamond–square color combination share homologous sequences. The designated ST and KL for each *A. baumannii* isolate are shown adjacent to their arrays. “*”: Low confidence in prediction. Isolates were grouped by CRISPR-Cas subtype: (A) I-F1, (B) I-F2, and (C) I-F2 containing two CRISPR arrays.

A total of 1,753 spacers, of which 338 were unique, were identified in the 34 CRISPR arrays of the 29 *A*. isolates. The average number of spacers per array was 51.6 ± 18.1. This number was higher for the arrays of the I-F1 systems compared to the I-F2 (60.2 ± 19.6 and 41.8 ± 9.9, respectively). The highest and lowest number of spacers were 84 and 18, respectively, with both arrays belonging to the I-F1 system. The spacer length was almost exclusively 32 bp, whereas the DRs were always 28 bp. The consensus DR sequences had 67.9% pairwise identity between the two subtypes: 5′-GTTCATGGCGGCATACGCCATTTAGAAA-3′ (I-F1) and 5′-GTTCTTCATCGCATAGATGATTTAGAAA-3′ (I-F2). A schematic representation of the spacers is illustrated in Fig. 4, and spacer information is found in Table S5. A high spacer homology was found within the same subtype, with a few exceptions, reflected by the number of arrays sharing the same diamond–square color combination (Fig. 4). Those isolates with identical CRISPR arrays (see CCRI19670, CCRI19672, CCRI19674, CCRI19675, CCRI19677) also had very high ANI (Fig. 1).

All spacers were blasted against three databases: the NCBI Virus database filtered for bacteriophages (42,648 nucleotide results and 168 RefSeq genomes), the PLSDB database for plasmids (50,554 sequences), and a prophage database (2,055 prophage sequences from 450 *A*. *baumannii* isolates), representing a total of 95,245 sequences. Of the 1,753 spacers, 889 (50.7%) had a matching sequence in at least one of the databases and against 1,646 different targets. The targeting spacers were distributed across the arrays, with no tendency to accumulate closer to the 5′ or 3′ end (Fig. S2), which indicates that they likely represent both recent and historical acquisitions, respectively (70). The number of matching sequences per spacer varied significantly, with spacers having only one target and others with over 500 targets, which can be attributed to the overrepresented conserved genes within prophage sequences. The spacers targeted 21 phages (1.3% of the 1,646 targets), which were either virulent, temperate, or filamentous, 133 plasmids (8.1%), and 1,492 *A*. *baumannii* prophages (90.6%). Of the 21 phages, 15 (71%) corresponded to *A. baumannii* phages. From the remaining targeted phages, they were from human gut metagenomes, one *Inoviridae* from an animal metagenome, and an *Erwinia* phage. Out of the 133 targeted plasmids, 129 (97%) were from *Acinetobacter* species, including among others *baumannii* (70), *seifertii* (13), *nosocomialis* (10), *pittii* (7), *indicus* (5), and *bereziniae* (4). Three different species from the *Edwardsiella* genus and one from *Agrobacterium* were also found, all Gram-negative bacteria. These results aligned with previous findings for *A. baumannii* CRISPR arrays in which, out of the spacers with identified matching sequences, a majority targeted temperate phages (24).

### Relationships between ARGs and CRISPR-Cas arrays

**Fig 5:**
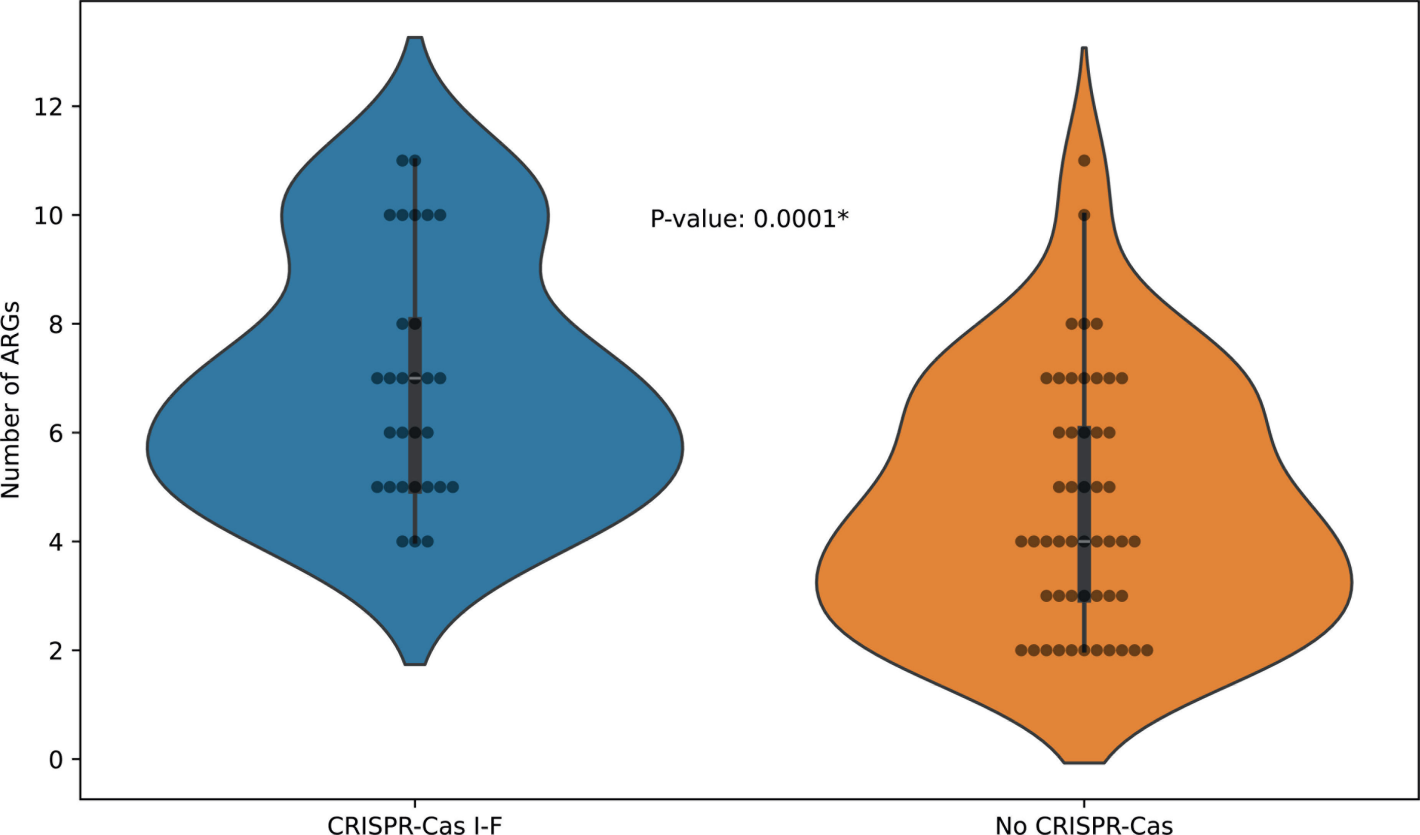
Distribution of ARGs in *A. baumannii* isolates with or without a subtype I-F CRISPR-Cas system. Violin plot depicting the number of ARGs in isolates with the presence of the subtype I-F CRISPR-Cas system (blue) and without any CRISPR-Cas system (orange). Mann-Whitney U test was performed to determine the *P*-value of 0.0001 (significant *P*-value < 0.05).

We compared our set of *A. baumannii* isolates in terms of the presence and absence of CRISPR-Cas systems with AMR and phage susceptibility (both *in vitro* and *in silico*). No significant correlation was found for most associations analyzed, except for the number of ARGs (Fig. 5). The distribution of ARGs shifted to a higher number of ARGs per isolate for those containing a CRISPR-Cas system (either I-F1 or I-F2), compared to the isolates lacking one. This is in contradiction with previous reports for this bacterial pathogen and others, as strains containing CRISPR-Cas systems tend to carry fewer antibiotic resistance genes (69, 71, 72). Others also observed that isolates carrying CRISPR-Cas systems lacked plasmids (73, 74), which supports the protective function of the CRISPR-Cas system against foreign invading DNA, like plasmids, and the acquisition of ARGs through mobile genetic elements (75–77). On the other hand, a previous *in silico* analysis reported that *A. baumannii* isolates carrying the subtype I-F CRISPR-Cas system had no association with resistance or virulence genes, with just a few exceptions. While no spacers were found to directly target these genes, they instead targeted plasmids (24). Yet, in another study, the presence of CRISPR-Cas systems did not correlate with the presence of ARGs in 104,947 reference genomes (from 5,677 bacterial species) (78). This would suggest that the CRISPR-Cas system would not hinder the dissemination of these genes, unless specifically plasmid-borne (78). In this study, we did not find any spacer targeting ARGs in plasmids according to the PLSDB metadata.

Collectively, this underlines that the mechanisms of action and regulation of CRISPR-Cas systems in relation to the acquisition of ARGs through mobile genetic elements are complex, and there are probably other factors like anti-CRISPR proteins or endogenous gene regulation yet to be defined (79). Moreover, while this CRISPR-AMR correlation was evidenced *in silico*, no significant correlation was found between the AMRs *in vitro*.

### Conclusion

In summary, this work has resulted in the creation of a well-characterized library of 79 clinical isolates of *A. baumannii*. The comprehensive *in vitro* and *in silico* analyses of these isolates enhance our understanding of this pathogen. The findings highlight the prevalence of certain international clones, though the factors contributing to their success need further investigation. Phage susceptibility in *A. baumannii* appears complex, with the KL serving as a good indicator of phage-bacteria interactions. Yet, the presence of CRISPR-Cas and other antiviral defense systems underscores their potential impact on these interactions. Moreover, we found that the CRISPR-Cas systems predominantly target prophages as observed by others (80, 81), potentially defending against latent threats if prophages enter the lytic cycle, promoting genomic stability, or contributing to the phage-bacteria coevolution. Future research should explore the diversity of isolates beyond clinical samples to gain a more comprehensive understanding of the population structure of *A. baumannii*. A more detailed characterization of bacterial and phage receptors, molecular mechanisms of lytic infection, as well as a deeper exploration of the defense mechanisms, will be needed for phage therapy to be successful against this critically important bacterial pathogen. Ultimately, these approaches will contribute to the development of novel therapeutic strategies against *A. baumannii* infections.

## Data Availability

Assembled draft whole genome sequences were submitted to the National Center for Biotechnology Information (NCBI) as BioProject PRJNA1081428. Accession numbers for each BioSample can be found in Table S1. Bacterial genomes were annotated during the NCBI submission using the Genome Annotation Pipeline (PGAP). Additional information is available on request from the corresponding author.

## SUPPLEMENTAL MATERIAL

Supplemental figures spectrum.02849-24-s0001.docx Fig. S1 and S2.

Supplemental tables spectrum.02849-24-s0002.xlsx Tables S1 to S8.

ASM does not own the copyrights to Supplemental Material that may be linked to, or accessed through, an article. The authors have granted ASM a non-exclusive, world-wide license to publish the Supplemental Material files. Please contact the corresponding author directly for reuse.
