# Supplementary material for: In silico and in vitro comparative analysis of 79 Acinetobacter baumannii clinical isolates
Source: Microbiol Spectr. 2025 May 16;13(7):e02849-24. doi: 10.1128/spectrum.02849-24 (PMC12210950; doi:10.1128/spectrum.02849-24)
Supplement: Supplemental figures — Fig. S1 and S2. [file spectrum.02849-24-s0001.docx]




**FIG. S1.** **Transmission Electron Microscopy (TEM) of phosphotungstic acid-stained phages**. A) Myoviruses, long contractile tails; B) Siphoviruses, long non-contractile tails; C) Podoviruses, short non-contractile tails.


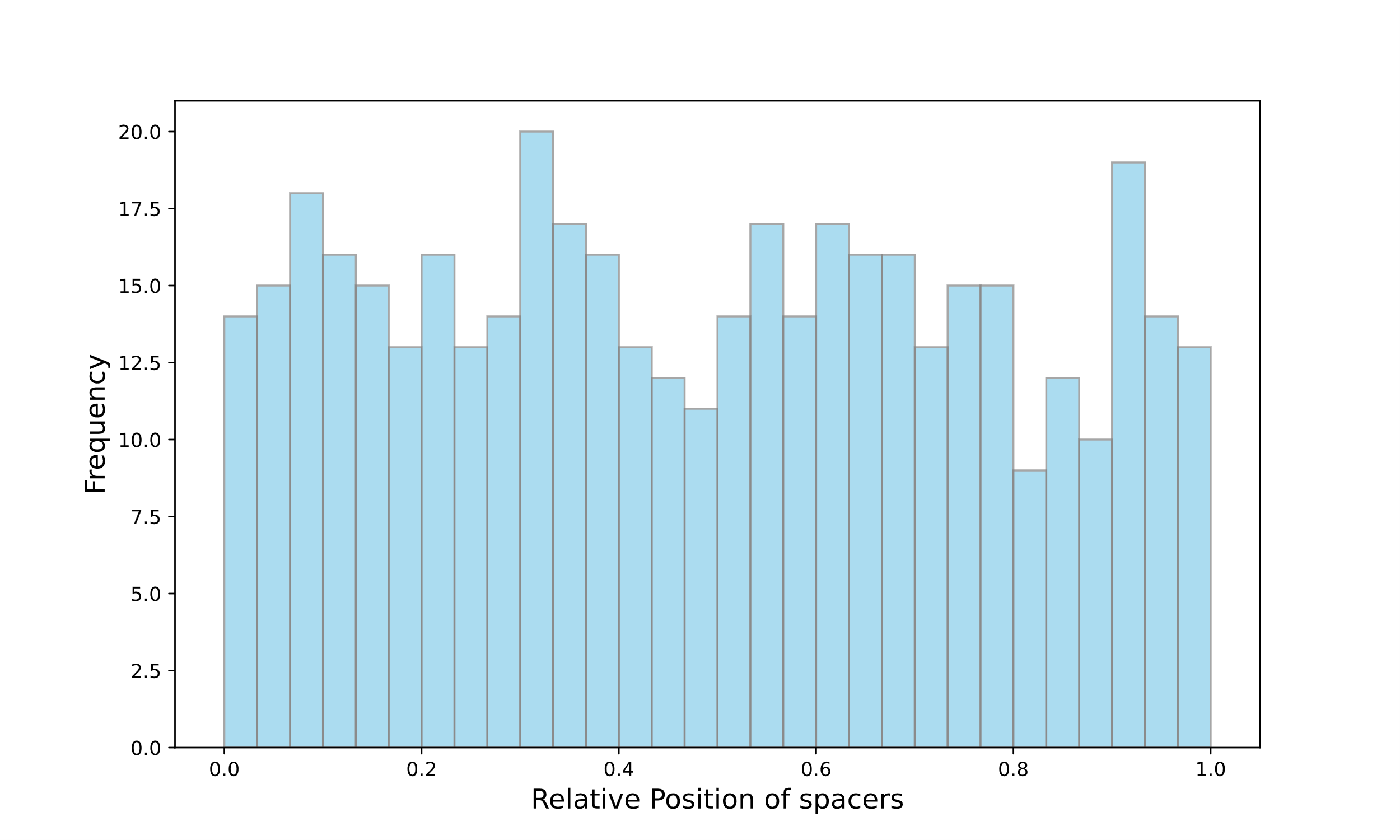


**FIG. S2. Relative position of spacers in the CRISPR arrays of the *A. baumanii* isolates**. The relative position of spacers is calculated considering the spacer position in the array and the total array length. Values closer to 1 are closer to the 3’ end and values closer to 0 are closer to the 5’ end in the array.
